# Staffing of Healthcare Workers and Patient Mortality: Randomized Trials Needed

**DOI:** 10.1371/journal.pmed.1001706

**Published:** 2014-08-19

**Authors:** Meeta Prasad Kerlin

**Affiliations:** Department of Medicine, Perelman School of Medicine at the University of Pennsylvania, Philadelphia, Pennsylvania, United States of America

## Abstract

In a Perspective accompanying a study by Benjamin Bray and colleagues, Meeta Kerlin discusses the evidence that physician and nurse workforces are associated with patient mortality, why clearer guidelines for appropriate workforce size are not available, and the next steps needed to address the knowledge gaps.

*Please see later in the article for the Editors' Summary*

**Linked Research Article**

This Perspective discusses the following new study published in *PLOS Medicine*:

Bray BD, Ayis S, Campbell J, Cloud GC, James M, et al. (2014) Associations between Stroke Mortality and Weekend Working by Stroke Specialist Physicians and Registered Nurses: Prospective Multicentre Cohort Study. PLoS Med 11(8): e1001705. doi:10.1371/journal.pmed.1001705

In a multicenter observational study, Benjamin Bray and colleagues evaluate whether weekend rounds by stroke specialist physicians, or the ratio of registered nurses to beds on weekends, is associated with patient mortality after stroke.

Sir Bruce Keogh, the Medical Director of the National Health Service, recently proposed a radical plan to overhaul urgent and emergency care services in the United Kingdom (UK) such that access to resources and services are equal across weekdays and weekends [1]. This seven-day services plan was based on evidence and belief that differences in weekend staffing and resource availability negatively affect patient experience and outcomes. It is one example of a global movement recognizing potential variability in care and outcomes during certain hours of the days, such as nights and weekends.

While it is hard to argue against the moral imperative to provide invariable quality of care round-the-clock, there is a necessary cost to providing maximum services at all times; therefore, understanding which resources and what staffing levels are minimally acceptable is also needed. Previous studies have yielded mixed results about off-hours care, suggesting that care during nights and weekends can indeed be provided with similar outcomes as that provided during weekdays [2]–[4]. However, there have been few rigorous studies to understand what those strategies are. In order to guide policies such as the NHS seven-day services model, the organization and processes that work must be examined.

In this issue of *PLOS Medicine*, Bray and colleagues have conducted a study that begins to address this issue [5]. They performed a prospective, multicenter cohort study of stroke units in the UK examining the associations between weekend rounding by stroke specialists and nursing staffing levels and patient outcomes. The primary findings were that the presence of seven-day-a-week rounding by a stroke specialist was not associated with a difference in mortality; however, the presence of a higher number of nurses per 10 beds was associated with reduced mortality, in a dose-dependent fashion. Although the study is observational in design, and therefore requires the cautious interpretation of non-experimental research, the authors performed robust analyses testing their assumptions with consistent results, providing some of the best evidence thus far of these associations.

## The Importance of Multidisciplinary Care

Much of the previous literature exploring the variability of quality of care and outcomes during off-hours has focused on physician staffing. These studies collectively have demonstrated that the effect of physicians differs depending on the setting. For example, in intensive care units (ICUs) without consistent daytime staffing by a critical care specialist, the presence of a nighttime specialist improves mortality [6]. On the other hand, in ICUs with specialists during the day, the presence of a nighttime specialist does not affect outcomes [6]–[8]. These variable findings raise the question of whether there are other ICU characteristics, besides the physician staffing model, that may affect patient outcomes.

In fact, patient care is multidisciplinary in nature, particularly that of the acutely and critically ill, such as those admitted to stroke units and ICUs. Prior evidence supports an association of multidisciplinary rounds with improved patient outcomes [9]. Bray and colleagues' results provide more evidence in favor of this notion: physician staffing did not affect patient outcomes by itself, whereas nursing staffing levels did. Their findings should prompt us to explore the importance of the role of not only nursing staffing, but of other disciplines and other organizational factors in off-hours care and outcomes.

## Facing the Challenge of Rigorous, Scientific Study of Nursing Staffing and Organization

Bray and colleagues' results add to an already rich body of literature from high-income countries indicating that higher levels of nursing staffing result in better outcomes [10]. Why then do no guidelines exist on minimum nurse-to-patient ratios intended to improve patient outcomes? Several factors synergize to perpetuate this policy gap. First, units in different hospitals with different case mixes have unique staffing needs, making a rule on staffing levels that can be generalized to different settings and countries almost impossible. Second, the evidence base is incomplete. Previous studies have consistently demonstrated that more nurses are better for patient outcomes, but no results have identified a “perfect” level. Furthermore, studies have been largely observational, and there have been no randomized trials to provide the sort of absolute evidence that might drive authoritative bodies to establish guidelines. Third, in the absence of an established minimum, individual units make individual decisions about staffing, taking into consideration perceived cost tradeoffs [11]. Last, in the face of ongoing shortages in the nursing workforce [12], staffing to any minimum level may be broadly infeasible.

This study is a call to research, a call to face the challenge to scientifically understand organizational factors that can inform policies such as the seven-day services model. Organizational research is by nature complex and difficult. Performing randomized trials of care processes and structural factors requires equipoise, funding, and buy-in from numerous stakeholder groups, any of which is often missing. However, the very factors that obstruct the existence of policies on nursing staffing are what makes them necessary. Knowing that nurses are in short supply, and that their greater presence is beneficial, the next steps must be taken to understand how best to allocate the limited workforce and what creative solutions can mitigate the effects of different staffing levels. Therefore, it is time to step up to the research challenges, prioritizing effort and funding to generate the same sound evidence base that is required of non-organizational factors that affect healthcare outcomes.

## Abbreviations

ICU: intensive care unit
